# The role of physics in multiomics and cancer evolution

**DOI:** 10.3389/fonc.2023.1068053

**Published:** 2023-03-17

**Authors:** Lucie E. Gourmet, Simon Walker-Samuel

**Affiliations:** ^1^ Centre for Advanced Biomedical Imaging, Division of Medicine, University College London, London, United Kingdom; ^2^ Centre for Computational Medicine, Division of Medicine, University College London, London, United Kingdom

**Keywords:** cancer, physics, evolution, multiomics, angiogenesis

## Abstract

Complex interactions between the physical environment and phenotype of a tumour, and genomics, transcriptomics, proteomics and epigenomics, are increasingly known to have a significant influence on cancer development, progression and evolution. For example, mechanical stress can alter both genome maintenance and histone modifications, which consequently affect transcription and the epigenome. Increased stiffness has been linked to genetic heterogeneity and is responsible for heterochromatin accumulations. Stiffness thereby leads to deregulation in gene expression, disrupts the proteome and can impact angiogenesis. Several studies have shown how the physics of cancer can influence diverse cancer hallmarks such as resistance to cell death, angiogenesis and evasion from immune destruction. In this review, we will explain the role that physics of cancer plays in cancer evolution and explore how multiomics are being used to elucidate the mechanisms underpinning them.

## Introduction

Cancer initially emerges from the accumulation of key mutations in somatic cells (1). The phenotypic changes that emerge from these mutations can provide a selective advantage over other cells that, through competition, can lead to clonal expansion (1). These processes are increasingly being studied and understood through an evolutionary lens (1). It has been established that tumours typically harbour a limited set of driver mutations that are positively selected through evolutionary pressures, alongside multiple passenger mutations that occur randomly (2). Likewise, deleterious (or disadvantageous) mutations are removed through negative selection (2). Cancer evolution has been shown to be dominated by positive selection and driver mutations have become an active area of cancer research (3). Meanwhile, negative selection affects cell survival and immune response (4). It is now becoming clear that a complex interplay exists between genetics and the physical properties of the tumour microenvironment, both of which can exert an influence in cancer evolution (5). This interplay can be seen as a two-way street: genetics and the physical environment are mechanistically coupled. This is reflected in the revised hallmarks of cancer in which physical mechanisms have been acknowledged such as phenotypic plasticity (6).

Biomechanics plays a key role in the development of cancer, such as *via* interactions between the extracellular matrix (ECM), cell membranes, cytoskeleton and blood vessels. This broad set of microstructural components are referred to as the physical microenvironment, and plays a fundamental role in the propagation of mechanical stress and fluid dynamics, both of which can have a direct impact on tumour growth and progression (7, 8). Here, we aim to summarise current understandings of the interaction between these physical properties and tumour ‘omics’ (genomics, transcriptomics, proteomics and epigenomics), alongside how they are incorporated into the emerging field of cancer evolution. We discuss the impact of physical constraints on cell fate and the spatial distribution of tumour cells. We finally focus on angiogenesis, and describe how this hallmark of cancer provides a good example of how the physical microenvironment and cancer biology intertwine.

## Biomechanical properties of tumours

### Mechanical stress, tumour proliferation and invasion

When external forces are applied to the surface of an object (e.g. a cell membrane), stress is defined as the resulting internal resistance to deformation (9). For a cell, the effect of stress depends on several factors, such as the duration of the force, its magnitude and direction, and the biomechanical properties of the cell and its surroundings. Stress directed towards the outside of a tumour can lead to disruption of the surrounding stromal tissue and an increase in ECM tension (10). Interestingly, in response to ECM stiffness, this tensile stress can result in the contraction of cellular actomyosin which can affect cell motility (10). Tensile stress appears to promote cell division, according to experiments in which collagen incisions lead to the relaxation of ECM tension and resulted in decreased cell invasion (11). These experiments therefore demonstrated that collagen contraction in the ECM induced by cancer cells can contribute to cancer invasion. In contrast, compressive forces can reduce cancer cell proliferation by limiting volume expansion (12). These forces can cause a decrease in cell volume, followed by an increase in the production of p27^Kip1^ which controls the cell cycle. As such, p27^Kip1^ therefore inhibits proliferation but in a reversible manner: after releasing mechanical stress, the number of cells overexpressing p27Kip1 decreases. External compressive forces can also cause blood vessels to be compressed, which can limit the delivery of nutrients to tumour cells, including oxygen, and result in regional hypoxia. Hypoxia can have multiple outcomes, depending on its severity and duration, including limiting progression (13), or, conversely, driving metastasis by contributing to angiogenesis and epithelial-to-mesenchymal transition (13). As indicated by this example, the interaction between mechanical stress and tumour proliferation is highly complex and multifactorial (13).

The propensity of a tumour to change in shape when exposed to mechanical stress is given by its stiffness (or its reciprocal property, elasticity). Elevated tissue stiffness has been reported in multiple cancers, including breast and brain, and was shown to promote cancer cell invasion by affecting pathways involving IDH1, a known cancer driver gene (5). Raised ECM stiffness is thought to be caused by an increase in protein deposition by cancer-associated fibroblasts, which are created from normal fibroblasts in response to carcinogenesis. The exact origin of cancer fibroblasts is unclear, but they are thought to emerge from the expansion of fibroblasts at the periphery of the tumour. ECM stiffness is further promoted through collagen crosslinking by lysyl oxidase and parallel reorientation of collagen fibres (9). Increased tissue stiffening has been shown to affect multiple other factors, such as cellular differentiation and vessel permeability (5). Moreover, experiments using a collagen matrix with graded directional stiffness showed that stiffness guides cell migration and directs cancer cells towards intravasation sites where new blood vessels are formed, which implicates the involvement of mechanical stress in cancer metastasis (14).

### The role of cell morphology and microarchitecture

**Figure 1:**
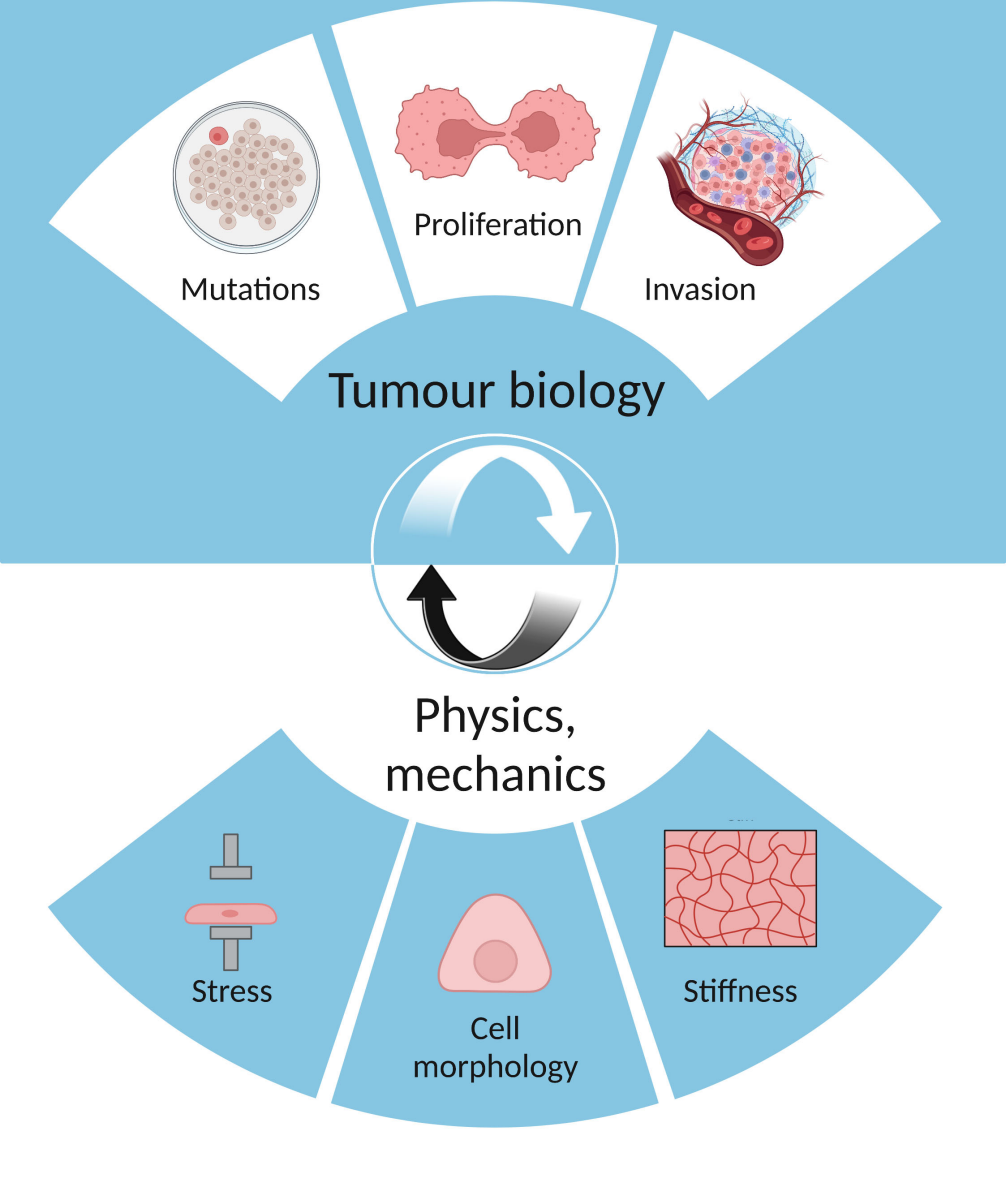
The interplay between tumour biology (mutations, proliferation, invasion) and physics (stress, cell morphology, stiffness).

The shape of a cell is determined by several biomechanical processes, alongside genetic influences (15). Likewise, cell shape and biomechanics can also impact the structure and function of tissue on a range of length scales (**Figure 1**). Cell shape also influences several pathological changes, as it has been shown to directly regulate proliferation, differentiation, and survival (15). For example, the localisation of the transcription factor NF-κB was found to be sensitive to cell–cell contact and cell area (15). Moreover, high levels of NF-κB in the nucleus were associated with having few cell neighbours and displaying a mesenchymal-type shape.

Cell morphology was also found to be moderated by specific oncogenes (Ras, Akt and Mek) and can distinguish early and late cancers (16). Using machine learning analysis of images of actin cytoskeleton and cell outline, they found morphological signatures of cancerous transformation. Interestingly, most shape measures recorded were useful parameters for cell classification, with an accuracy ranging from 69.5% to 97.5%. Similar shape changes were associated with cancer progression across different cell types (osteoblastoma, breast cancer, retina). Therefore, this study not only shows that cellular morphology reflects the ‘internal’ state of a cell, but it also illustrates how this interplay can be exploited to better understand cancer evolution. Another study investigated the difference in morphological behaviour between metastatic and healthy cells (17). They developed a potential tool for early cancer detection which confirms the fact that features involving cell geometry and cellular protrusions help distinguish normal from cancer cells.

Mechanics has a further role in cellular organisation, for example in phase separation. Phase separation is the process responsible for cellular compartmentalization, involving the assembly of condensates which are membraneless organelles such as stress granules and polycomb bodies (18). Dysregulated components in cancer can affect the location and the regulation of condensates, thereby leading to altered gene expression (19). Thus, this example demonstrates a physical process affecting the evolution of cancer and of multiomic factors.

## The influence of physical forces on cancer progression

### Nuclear deformation and genome integrity are closely linked

Cells are mechanosensitive which means that they can respond to applied forces *via* cell-cell/cell-ECM adhesions and ion channels that are sensitive to stress (20). However, few studies have investigated whether biomechanical properties directly influence mutational burden. Nuclear pore complexes modulate the import of transcription factors when nuclei are under solid stress and deformed (21). Nava et al. found that the genome is protected from mechanical stress, as nuclear deformation is counteracted *via* a calcium-dependent nuclear softening driven by loss of H3K9me3-marked heterochromatin (22). This chromatin mechanoprotection is significant as it prevents large-scale genetic changes, such as chromosomal breaks or rearrangements, and means that chromosome condensation can be modified to adapt to physical pressures. Less condensed chromosome regions enable gene expression, which positions physical stress as a factor that directly affects transcription. Thus, this protection mechanism is important for both genome integrity and gene expression. Moreover, increased ECM stiffness has been shown to induce DNA damage in mammary epithelial cells through reactive aldehyde species (23). They showed that DNA damage accumulation was due to a decrease in the clearance of these reactive aldehyde species and downregulation of aldehyde dehydrogenases, which is known to counteract oxidative stress. ECM stiffness was also reported to drive genomic heterogeneity in MYCN-amplified neuroblastoma cell lines as distribution of ECM tension is translated into genotypic variations *via* mechanotransduction, the ability of transforming mechanical signals into biological signals (23). This implies that the mutational status of a tumour in part reflects the state of the tumour microenvironment, just as the microenvironment reflects mutational status. Thus, this further highlights how the interplay between the internal state of the tumour and the physical environment is bi-directional.

### Transcription is responsive to the physical environment

Fluid shear stress disrupts genome integrity and can also lead to changes in gene expression. Tajik et al. demonstrated that the stretching of chromatin *via* an external force result in transcription upregulation (24). The stress angle of the material used to apply pressure on the surface of a cell determines the extent of chromatin stretching and consequently affects the magnitude of DHFR transcription upregulation. Several models have been proposed to explain how mechanotransduction systems directly affect transcription, such as YAP (Yes-associated protein) and TAZ (transcriptional coactivator with PDZ-binding motif) translocating from the cytoplasm to the nucleus. Dupont et al. showed that YAP/TAZ transcriptional activity is regulated by ECM stiffness by growing mammary epithelial cells on cells on ECM of high versus low stiffness and analysing their gene expression (25). They also found that YAP/TAZ depends on cell geometry as the localisation of YAP/TAZ changed from predominantly nuclear in stiff ECM, to predominantly cytoplasmic in cells in soft ECM. Conversely, YAP/TAZ levels affect cell behaviours such as apoptosis (25). These findings concur with other research which found that YAP/TAZ respond to cell crowding and that cells under tensile stresses activated YAP/TAZ thereby leading to cell proliferation (26). Under stiff ECM conditions, the activation of YAP and TAZ transcription is dependent on actin and integrin, molecules that are associated with movement (27). Integrin also has a direct role in cancer gene expression as αvβ3 integrins were found to induce the transcription of the proapoptotic gene PUMA (p53-upregulated modulator of apoptosis) which promotes tumour stemness (28). Overall, these studies describe a direct link between transcriptomics, genomics and the physics of the tumour microenvironment.

### Epigenetic processes are regulated by fluid dynamics and stiffness

**Figure 2:**
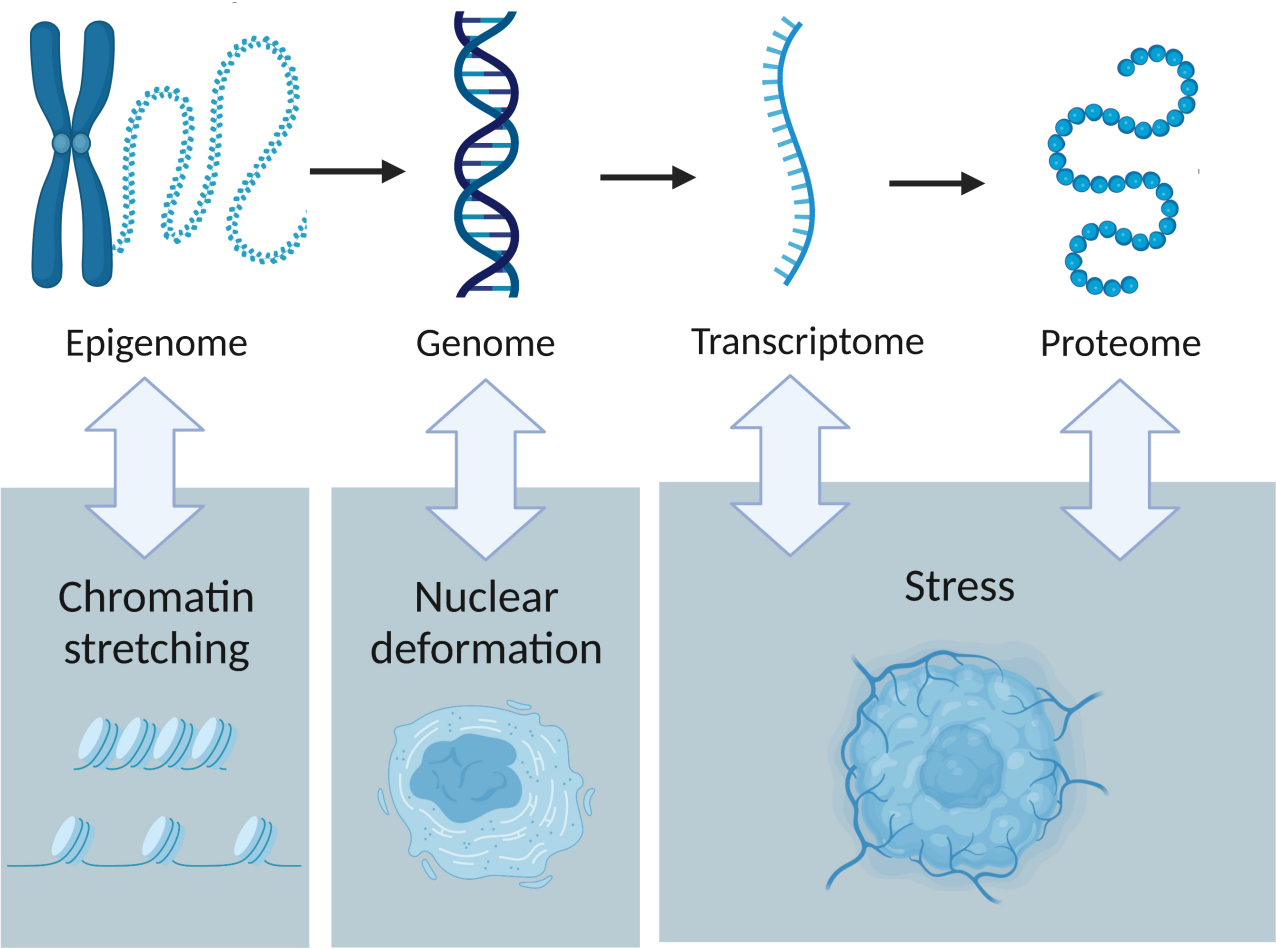
The interactions between tumour omics and the physical traits of cancer. Epigenetics is influenced by chromatin stretching, nuclear deformation leads to genome instability and stress affects both transcription and protein production.

Epigenetics refers to heritable or environmental changes in gene expression which are not due to mutations. For example, DNA methylation, histone modifications and RNA mechanisms alter gene expression without modifying the DNA sequence itself. Haemodynamic forces including laminar and oscillatory flow, and cyclic strain have been shown to induce epigenetic modifications in vascular cells (29). For example, shear stress can modulate chromatin remodelling on histone H3 and H4 which appears to control the expression of endothelial nitric-oxide synthase (30). This finding shows that specific histone modifications have an important role in vascular gene expression, thereby affecting blood vessel formation in a pathological context such as cancer. Moreover, the endothelial-specific microRNA miR-92a was shown to promote fluid flow-stimulated angiogenesis but its expression is induced by the mechano-sensitive zinc finger transcription factor klf2a (31). This microRNA facilitates angiogenic sprouting and is dependent on mechanical factors. Epigenomic studies showed that ECM stiffness drives epigenetic changes with experiments using mechanosensitive breast cancer 3D culture model (32). Stowers et al. demonstrated that stiff matrices generated an increase in nuclear wrinkling compared to cells in soft matrices. In stiff matrices, heterochromatin condensed accumulations were found at the nuclear periphery and heterochromatin thickness increased compared to soft matrices, indicating that chromatin state was broadly misregulated. Their experiment showed the direct impact that stiffness has on chromosomal structure and gene expression. Interestingly, these chromatin changes were associated with a tumorigenic phenotype in the mammary epithelium which highlights the importance of the interplay between epigenomics and tumour mechanics (**Figure 2**).

## The consequences for tumour evolution

### Physical changes affect cell fate

The physical traits of cancer arise through conventional biological processes, but the reverse is also true; mutations, transcription, protein expression and epigenetics are each affected by the physical characteristics of the microenvironment. For example, ECM remodeling is due to the activation of matrix metalloproteinases, which are initially inert due to the interaction between the pro-domain and the catalytic site of these proteins (33). However, physical alterations in cancer can disrupt this interaction and enable migration and metastasis (33).

Epithelial-mesenchymal transition (EMT) is the process in which epithelial cells become mesenchymal cells through the loss of cell polarity and adhesion. This transdifferentiation has been implicated in cancer as mesenchymal cells are more motile, which enables invasion and metastasis. EMT is also affected by the physical traits of tumours, as ECM stiffness promotes EMT *via* a EPHA2/LYN complex and a TWIST1-G3BP2 pathway (34, 35). In addition, tissue geometry has been shown to have an impact on EMT, as changing the shape of the epithelial sheet altered the spatial pattern of EMT and EMT-permissive regions were found to experience the highest mechanical stress (36). Cancer stem cells, which have been linked to therapy resistance and cancer recurrence, are also subject to the mechanical forces of the tumour microenvironment. ECM stiffness increases significantly from the centre outwards, which has implications for the understanding of cancer stem cell development (37). Indeed, the most invasive and metastatic cancer stem cells were found to be mainly concentrated in the invasive outer edge of a tumour (38). The microenvironment of this area enables the clonal expansion of the cancer stem cells present (38). Furthermore, hydrodynamic shear stress was found to promote the conversion of circulating tumour cells to cancer stem-like cells in the blood circulation *via* metabolic reprogramming (glycolysis and amino acid exchange) from the tumour niche (39). Thus, tumour mechanics specific to blood vessels provide an advantage to the circulating cells as they gain new oncogenic properties. This finding supports the fact that the location of a cancer cell matters because its environment can affect its behaviour.

### Spatial context matters for cancer progression

Many studies have used multi-omics approaches to study cancer progression but Srivastava et al. uncovered salient features of both imaging and omics data (40). They compared the performance of transcriptomics, proteomics and tissue images in predicting four domains: tumour stage, oestrogen receptor status, American Joint Committee on Cancer (AJCC) staging and PAM50 subtype. They found that histology images were best for predicting AJCC and tumour stage while transcriptomics data was better at characterising Estrogen Receptor (ER) status and PAM50 subtypes. The ability to compare imaging data with multi-omics data is important because it shows that some methods are more relevant based on the aim of a study. This type of study is interesting, but it requires having both imaging and sequencing data from the same patient, which is rarely the case.

The architecture of normal tissue has been shown to influence the fitness of cancer cells and determine the mode of evolution as a result of competition for space (41). In fact, this study showed that spatial limitations and cell mixing rates are the main factors influencing the acquisition of cancer driving mutations. Thus, the outcome of a selective advantage at a cellular level depends on the environmental competitive context, which highlights the importance of spatial constraints. The different modes of tumour growth (surface or volume) were found to impact the extent and the distribution of clonal diversity over time (42). Interestingly, this study reveals that tumour roundness may be representative of clonal diversity: when the tumour from their cellular automaton model (representing clear cell renal cell carcinoma) grows, it eventually loses its round shape but recovers it mirroring the increase and decrease of clonal diversity during tumour evolution. Noble et al. also showed that spatial structure governs the mode of tumour evolution by analysing four types of tissue structure (non-spatial, gland fission, invasive glandular, and boundary growth) (43). The different tumour architectures influenced the distribution of mutation frequency and the dynamics of clonal diversity. For example, hepatocellular carcinoma (whose architecture is described as boundary growth, where proliferation is confined to the boundary) was found to promote genetic drift and was subject to a mutation burden increasing from the tumour core to its boundary. For invasive glandular tumours (which represent most solid tumours), small increases of cell fitness are sufficient to trigger a clonal expansion. The evolution of invasive glandular tumours can be described as a branching process, where mutations create multiple new clones without necessarily removing old clones. The conclusion from these findings is that alterations of tumour architecture during cancer do impact the mode of tumour evolution.

Because physical and spatial characteristics of tumours affect cancer evolution, they are interesting in the context of multi-omics. To enrich our current understanding of cancer, we can investigate the interplay of different cancer hallmarks, such as angiogenesis and genome instability. In this example, one could wonder whether blood vessels impact the mutational diversity of a tumour. Angiogenesis provides nutrients to cancer cells and enables the circulation of cancer cells which makes it worth investigating.

## The interplay between angiogenesis and tumour evolution

The interaction between blood vessels, tumour biomechanics and genomics is highly complex, which makes it challenging to investigate. However, angiogenesis offers a useful system to highlight the complex relationship between genetics and physical microenvironment.

Blood vessel formation in cancer occurs in response to the need for nutrients and oxygen to sustain homeostasis and growth. Sherwood et al. found that rapidly growing tumours are dependent on angiogenesis whereas dormant tumours are not heavily vascularised (44). They also showed that inhibition of angiogenic signalling pathways prevents vessel formation and leads to tumour dormancy (45). The first step in tumour vascularisation is the angiogenic switch, during which pro-angiogenic signalling such as VEGF becomes dominant over anti-angiogenic factors (46). Blood formation can be initiated in several ways, including sprouting angiogenesis, intussusceptive angiogenesis, vasculogenesis, endothelial progenitor cells, vasculogenic mimicry and transdifferentiation of cancer cells (47). These mechanisms result in very different vessel morphologies, with differing flow and delivery characteristics, and can therefore provide a range of environments for cancers to evolve within. Certain cancer types have corresponding characteristic vascular phenotypes: intussusceptive angiogenesis has been observed in melanoma and adenocarcinoma, while vasculogenesis has been reported in gliomas (47). For example, vascular mimicry has been linked to aggressive tumours and reported following anti angiogenic therapy, making it an alternative neovascularisation process used to survive treatment. It also has been associated with poor prognosis in colorectal cancers and glioblastomas (47). The different types of angiogenesis are important regarding treatment options as it was shown that inhibition of vasculogenesis prevents glioma recurrence, not sprouting angiogenesis (48).

### Blood vessels impact tumour heterogeneity

**Figure 3:**
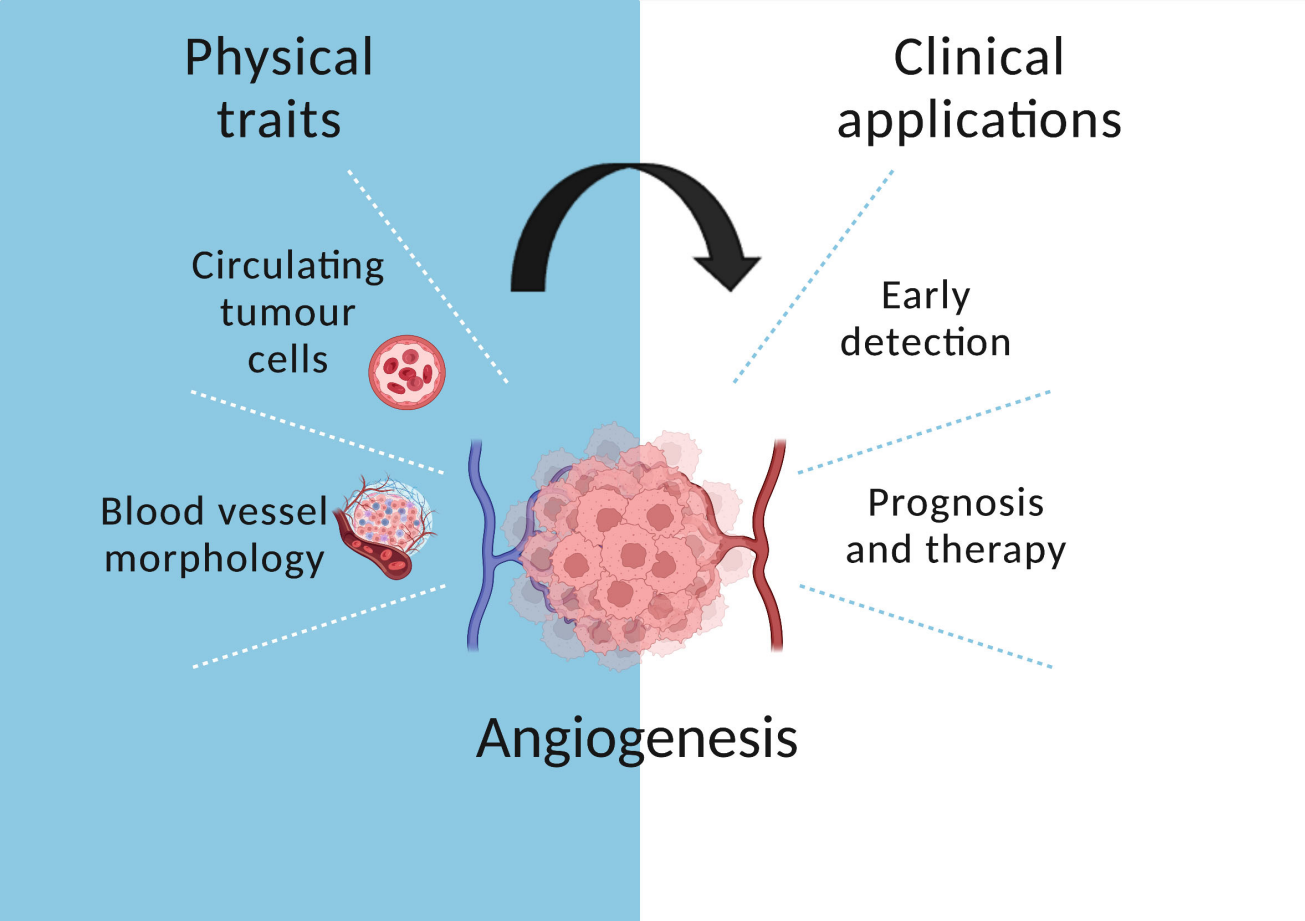
The role of angiogenesis in cancer evolution. Circulating tumour cells are released in the bloodstream which enables early detection. In addition, blood vessel morphology informs prognosis and treatment.

Computational simulations of the microenvironment of tumours showed that small tumours are spatially heterogeneous when vascularised (49). This finding implies that blood vessels lead to high evolutionary pressure and selection of different clones depending on the microenvironment even in an early cancer stage, when the tumour is still small. Moreover, Sartakhti et al. used an economic model to simulate angiogenesis, where the secretion of pro-angiogenic factors were considered to be public goods that can be exploited by free-riders (i.e. cells that cease to contribute to angiogenesis formation) (50). In this approach, a clone’s fitness is determined by collective interactions and a critical mass of cells cooperating is necessary to sustain angiogenesis. Their results revealed that spatial heterogeneity may be a main driver underlying the emergence of angiogenic clones and showed that sprouting of new blood vessels from surrounding vascular tissue is directed towards the centre of the tumour. This is in accordance with studies which already reported this pattern in Lewis lung carcinoma and mammary carcinoma (51, 52). The architecture and morphology of angiogenesis is therefore critical in cancer evolution and has important implications for cancer treatments (**Figure 3**).

### Timing is crucial for angiogenic morphology

In addition to spatial heterogeneity, blood vessels are also dependent on time-based properties as Bentley and Chakravartula highlighted that endothelial cell rearrangements are regulated by the speed of selection (53). Vessel sprouts are generated in response to hypoxia, with leading tip cells inhibiting stalk cells and being selected to lead sprout growth by a central pattern generating (CPG) mechanism involving Dll4-Notch lateral inhibition in a feedback loop with VEGF – VEGFR signalling (53). Slowing down the CPG mechanism, meaning that cells take longer to decide on their movement, leads to a sparser branching phenotype. Synchronised selection switches vessels from branching to expansion and accelerating selection preserves sprout extension in proliferative tissues. Thus, tissue-derived factors such as VEGF, and active perception *via* filopodia extensions can vary the speed and timing of cell migration. Temporal variability has many implications, including choosing a convenient targeting time for therapeutic normalisation. The environmental conditions are important as well since they influence the eco-evolutionary causes of temporal changes in angiogenesis (54). In this review, angiogenesis is assimilated to an ecoevolutionary process called niche construction and is compared to organisms improving their environment, for example dams constructed by beavers. Temporal fluctuations are thought to either promote a plastic phenotype or select for speciation. In the former case, the cancer cell is viewed as a jack of all trades which can adapt to different microenvironments or treatments easily. On the other hand, cancer cells can specialise through foraging and metabolism. These adaptive strategies are subject to trade-offs which are common in nature, which is why eco-evolutionary dynamics are studied to get a better understanding of cancer.

## Conclusion

Genomics is fundamental to the understanding of cancer evolution, but tumours are also subject to mechanical, compressive, tensile and shear stresses that can play a significant role. For example, raised interstitial fluid pressure can promote cancer cell invasion in the surrounding tissue, and matrix stiffness differs between the centre and the border of the tumour, which helps metastasis by guiding cell migration. Cell geometry can inform on cell function, indicate whether specific mutations are present and predict malignancy. These mechanical characteristics form a complex dynamic system, with interactions between physical features, genetic mutation and expression, epigenetics and protein transcription. Stiffness and shear stress have been shown to lead to DNA damage. The transcription of YAP and TAZ is dependent on tensile stress, and chromatin stretching was shown to upregulate transcription. In addition to the structure of chromatin, epigenetic modifications are essential as shear stress was found to promote angiogenesis by affecting histones.

Computational models and simulations help us predict the impact of tissue architecture, but quantitative imaging analysis is needed to confirm their hypotheses. The physical and omic interactions matter for cancer evolution because they alter cell behaviour, facilitating the development of stem cells and mesenchymal cells. It also helps the circulation of tumour cells by increasing tumour shedding and enhancing angiogenesis. Angiogenesis plays a pivotal role as the mechanism underlying blood vessel formation leads to diverse morphologies and is subject to temporal variations influencing cancer prognosis and therapy. We can view cancer as an ecological system and use strategies found in nature to model cancer evolution. Investigating the impact of angiogenesis on other cancer hallmarks such as mutations and immune evasion will provide a deeper understanding of this disease. Moreover, studying angiogenesis is useful to elucidate the interplay between physics and biology. To improve our current detection methods, we need to continue interdisciplinary research to understand how these diverse aspects of cancer fit together.

## Author contributions

LG conducted the literature review and SW-S provided feedback, reviewed the article. All authors contributed to the article and approved the submitted version.
